# Dietary Avian Proteins Are Comparable to Soybean Proteins on the Atherosclerosis Development and Fatty Liver Disease in *Apoe*-Deficient Mice

**DOI:** 10.3390/nu13061838

**Published:** 2021-05-27

**Authors:** Roberto Martínez-Beamonte, Javier Sánchez-Marco, Gonzalo Lázaro, María Barco, Tania Herrero-Continente, Marta Serrano-Megías, David Botaya, Carmen Arnal, Cristina Barranquero, Joaquín C. Surra, Jesús Osada, María A. Navarro

**Affiliations:** 1 Departamento de Bioquímica y Biología Molecular y Celular, Facultad de Veterinaria, Instituto de Investigación Sanitaria de Aragón, Universidad de Zaragoza, E-50013 Zaragoza, Spain; romartin@unizar.es (R.M.-B.); donjaviersanchez@gmail.com (J.S.-M.); gonzalolbe98@gmail.com (G.L.); m.barco.campos@gmail.com (M.B.); taniaherrero1992@gmail.com (T.H.-C.); cbarranq@unizar.es (C.B.); angelesn@unizar.es (M.A.N.); 2 Instituto Agroalimentario de Aragón, CITA-Universidad de Zaragoza, E-50013 Zaragoza, Spain; arnal@unizar.es (C.A.); jsurra@unizar.es (J.C.S.); 3 CIBER de Fisiopatología de la Obesidad y Nutrición, Instituto de Salud Carlos III, E-28029 Madrid, Spain; 4 Aves Nobles y Derivados-Aldelis, E-50197 Zaragoza, Spain; mserrano@aldelis.com (M.S.-M.); dbotaya@aldelis.com (D.B.); 5 Departamento de Patología Animal, Facultad de Veterinaria, Instituto de Investigación Sanitaria de Aragón, Universidad de Zaragoza, E-50013 Zaragoza, Spain; 6 Departamento de Producción Animal y Ciencia de los Alimentos, Escuela Politécnica Superior de Huesca, Instituto de Investigación Sanitaria de Aragón, Universidad de Zaragoza, E-22071 Huesca, Spain; 7 Department of Biochemistry and Molecular Biology, Veterinary School, University of Zaragoza, Miguel Servet, 177, E-50013 Zaragoza, Spain

**Keywords:** dietary protein, chicken, turkey, soybean, atherosclerosis, *Apoe*-deficient mice, paraoxonase

## Abstract

Background and aim: The type and amount of dietary protein has become a topic of renewed interest in light of their involvement in metabolic diseases, atherosclerosis and thrombosis. However, little attention has been devoted to the effect of avian proteins despite their wide human consumption. The aim was to investigate the influence of chicken and turkey as sources of protein compared with that of soybean on atherosclerosis and fatty liver disease. Methods and results: To this purpose, male and female *Apoe*-deficient were fed purified Western diets differing in their protein sources for 12 weeks. After this period, blood, liver, aortic tree and heart base samples were taken for analyses of plasma lipids and atherosclerosis. Plasma triglycerides, non-esterified fatty acids, esterified cholesterol levels and radical oxygen species in lipoproteins changed depending on the diet and sex. Females consuming the turkey protein-containing diet showed decreased atherosclerotic foci, as evidenced by the en face atherosclerosis analyses. The presence of macrophages and smooth muscle cells in plaques were not modified, and no changes were observed in hepatic lipid droplets in the studied groups either. Paraoxonase activity was higher in the group consuming turkey protein without sex differences, but only in females, it was significantly associated with aortic lesion areas. Conclusions: Compared to soybean protein, the consumption of avian proteins depending on sex resulted in similar or lower atherosclerosis development and comparable hepatic steatosis.

## 1. Introduction

The ingested dietary protein is a crucial target related to several diseases. US government dietary guidelines recommend a protein intake between 10 and 35% of the total caloric value (TCV) for maintenance and growth, with a current consumption of the population around 16% of TCV [1]. It is important to note that not all the ingested protein is absorbed, and that proteins are composed of 20 amino acids, 9 of which are essentials in humans and other animals, and must be obtained directly from the diet. For this reason, a suitable proportion and amount of every essential amino acid for a well-balanced diet is important. In 2013, The Food and Agriculture Organization (FAO) published a reference manual to evaluate nutritional protein quality [2], which incorporated aspects such as digestibility, considering the digestibility of individual amino acids to assess their availability as digestible indispensable amino acid score (DIAAS). Knowing the proportion of available amino acid of protein source is crucial to fulfil the different physiological requirements for maintenance and/or growth and according to each species [3,4].

The influence of source of proteins modulating the predisposition to certain pathologies has received little attention when compared to other macronutrients, once the physiological protein needs of an animal are met. The fact that amino acids may require specific transporters and some receptors are sensitive to these compounds are elements to hypothesize that they could contribute to the regulation of proteostasis and through an imbalance of the latter to the development of diseases [5]. Recent papers have reinforced this notion, the impaired metabolism of branched-chain amino acids (BCAA), that include the three essential amino acids leucine, isoleucine and valine, promotes thrombosis risk by platelet activation induced by the valine and isoleucine metabolites [6]. In vitro incubation of endothelial cells in presence of BCAA resulted in endothelial dysfunction through influencing oxidative stress [7].

The best way to evaluate the influence of the dietary amino acid composition of a certain protein, keeping the rest of the nutrients unchanged, is achieved by the use of purified diets [8] when using the mouse lacking apolipoprotein E. The latter develops spontaneous atherosclerosis and fatty liver in a short period of time, and for that reason, it is considered a good animal model to test the influence of nutrients on these ailments [9,10]. Using this model, a decreased atherosclerosis by administration of soybean protein has been reported with independence of lipoprotein changes [11,12], with the β-conglycinin component of soy being a particularly active protein [13]. In addition, other non-protein components of soybean such as isoflavones have shown anti-atherosclerotic properties as well [14,15]. Little attention has been paid to avian sources of proteins and in this regard, chicken protein was found similar to soy protein considering atherothrombosis in mice [16]. When compared with the control diet, chicken collagen hydrolysate was able to reduce atherosclerosis associated with inflammatory markers in *Apoe*-deficient mice [17]. No study has addressed the effect of turkey proteins on atherosclerosis. High variability in amino acid contents has been observed in the composition of soybean, chicken and turkey proteins among different studies [18,19,20,21], the only aspect that seems to be independent of studies is the ratio of essential/non essential amino acids that was lower in the soybean than in the avian proteins. Based on these statements, it could be hypothesized that turkey and chicken proteins might display similar biological effects assuming their avian phylogeny. The present study was designed to compare the effects of those avian proteins with the soy protein on the development of atherosclerosis and fatty liver in *Apoe*-deficient mice of both sexes. A characterization of diet amino acid composition of these diets was done to verify whether observed changes could be ascribed to amino acid composition differences.

## 2. Material and Methods

### 2.1. Animals and Diets

*Apoe*-deficient mice on C57BL/6J genetic background were obtained from Charles River (Charles River Laboratories, Barcelona, Spain) and bred at our animal facility. To establish groups with similar initial weight and plasma cholesterol, 45 male and 30 female, two-month-old mice were weighed, blood samples taken (after four-hour fasting) from the facial vein and their cholesterol analyzed. Six groups of *Apoe*-deficient mice were allocated, 3 groups for males and other 3 for females and housed in sterile filter-top cages in rooms maintained under a 12-h light/12-h dark cycle in the Centro de Investigación Biomédica de Aragón. All had ad libitum access to food and water. Mouse experiments were carried out in accordance with the EU Directive 2010/63 on the protection of animals used for scientific purposes and the study protocol was approved by the Ethics Committee for Animal Research of the University of Zaragoza (PI36/18).

During the 12 weeks, mice received a Western feed-regime, based on the purified AIN-93 diet for laboratory mice [22], supplemented with a 20% of palm oil and 0.15% of cholesterol, but replacing the original casein for one of the different sources of purified protein, soybean, chicken or turkey origin, as shown in Table 1. Soy protein isolate was purchased from Sinoglory Enterprise Group (Qingdao, China) and chicken and turkey proteins were prepared removing all visible fat, boiled, crushed and lyophilized (Aldelis, Zaragoza, Spain). Analyses of chicken and turkey protein preparations showed triglyceride contents lower than 0.5% equivalent to the other protein source. All diets were prepared in our facilities, lyophilized and stored at −20 °C in vacuum bags until use. An aliquot was subjected to nitrogen content and amino acid composition analyses.

Intake and body weights were monitored every 2 weeks. At the end of the 12-week dietary intervention, food was withdrawn for 12 h, and the mice were weighed and then sacrificed by suffocation in a CO_2_ chamber. Blood samples were drawn by cardiac puncture, and plasma and serum were centrifuged at 3000× *g* for 10 min. The livers were removed and frozen in liquid nitrogen and stored at −80 °C until processing and an aliquot was stored in buffered formaldehyde. Heart and aorta were perfused with PBS, hearts were filled with OCT Tissue-Tek^®^ (Sakura Finetek, Barcelona, Spain), frozen in dry ice and stored at −80 °C, while dissected aortas were kept in buffered formaldehyde at 4 °C.

### 2.2. Dietary Characterization

Nitrogen content was measured by the Kjeldahl method [23]. Amino acid composition of the diets was quantified according to the recommended methods of analyses [24].

### 2.3. Plasma Determinations

Total plasma cholesterol and triglyceride concentrations were measured in a microtiter assay, using Infinity^TM^ commercial kits (Thermo Scientific, Madrid, Spain), glucose (BioSystems, Barcelona, Spain) and non-esterified fatty acids (NEFA) (Fujifilm Wako chemicals, Richmond VA, USA) according to the manufacturer’s instructions. Total serum apolipoprotein A1 (APOA1) was quantified by ELISA [25] and arylesterase activity of paraoxonase (PON1) as previously described [26]. Plasma lipoprotein profile was determined in 100 μL of pooled plasma samples from each group and sexes by fast protein liquid chromatography (FPLC) gel filtration using a Superose 6B column (GE Healthcare, Chicago, Il, USA) in 48 fractions as previously described [27].

### 2.4. Reactive Oxygen Species (ROS) Content in Lipoproteins

The presence of ROS was assessed by measuring the conversion of 2,7-dichlorofluorescein diacetate into fluorescent dichlorofluorescein [28] in FPLC-isolated fractions corresponding to the different lipoproteins [29].

### 2.5. Evaluation of Atherosclerotic Lesions

En face analyses of aortas from the heart to their iliac bifurcations were carried out. Basically, they were soaked in Oil Red O Stock solution (35 mL of 0.2% (*w/v*) Oil Red O in methanol mixed with 10 mL of 1 M NaOH and filtered) for 50 min, rinsed in 78% of methanol for 5 min [30] and cleaned of all external fat. Then, aortas were cut open longitudinally, and stained with Oil Red O Stock solution to ensure complete staining of all internal lesions. The images were captured with a Canon EOS 600D with a Sigma 105 mm macro lens mounted in a Kaiser RA1 arm and blindly analyzed with Adobe Photoshop CS2 (Adobe Inc. San Jose, CA, USA).

For cross-sectional analyses, the OCT-embedded aortic bases of the dissected hearts were frozen and cut into 5-μm slices. Some serial cryosections of the proximal aorta and the aortic sinus were stained with Sudan IV B (Sigma Chemical Company), and counter-stained with hematoxylin and eosin (Sigma Chemical Company) as previously described [31]. Lesion sizes were used for morphometric evaluations based on the method developed by Paigen et al. [32]. Images were captured using a Nikon microscope equipped with a Canon digital camera. Morphometric analyses were also blindly evaluated using Adobe Photoshop CS2.

In order to characterize lesions, other cryosections were fixed for 5 min in acetone and kept frozen until use. After thawing, the sections were rehydrated in PBS for 5 min and blocked with 5% BSA containing PBS for 1 h at room temperature and immunostained in two different ways. First, macrophage fluorescent immunostaining was carried out using a rat anti mouse CD68-Alexa Fluor^®^ 488 (MCA1957A488, AbD Serotec, Oxford, UK- diluted 1/100 in 2.5% BSA/PBS) by incubation overnight at 4 °C. Then, the sections were washed three times with 1% Tween-20 in PBS and mounted with ProLong™ Diamond Antifade Mountant with DAPI (P36966, Thermo Fisher Scientific). Next, 20X Fluorescent images were captured by ZEISS Axio Scan.Z1 Slide Scanner and CD68 areas were quantified using Zeiss ZEN Lite o Zeiss ZEN (Blue edition) analysis software. Second, presence of smooth muscle cells (SMC) was evaluated by immunohistochemistry of cryosections using an anti-mouse -actin conjugated to alkaline phosphatase (Sigma Chemical, Madrid, Spain, diluted 1:25). Non-specific binding was removed by repeated washes and bound alkaline phosphatase activity was revealed with Fast Red TR/ Naphtol AS-MX (Sigma Chemical, Madrid, Spain) as substrate. Visualization of red color indicated presence of SMC. Images were captured as described above and data are expressed as surface of pixels occupied by SMC in fibrous caps.

### 2.6. Hepatic Histological Analyses

Paraffin sections (4 μm) from the livers stored in formaldehyde were stained with hematoxylin and eosin, and a slide scanner Zeiss AsioScan.Z1 (Zeiss, Oberkochen, Germany) was used to capture all preparations. Lipid droplets were evaluated quantifying their extent in each liver section with Adobe Photoshop CS3 and expressed as percentage of total liver section as previously described [31].

### 2.7. Statistical Analyses

Sample size calculations accepting an alpha risk of 0.05 and a beta risk of 0.2 in a one-sided ANOVA test of 3 groups were calculated based on the atherosclerotic parameters with the highest standard deviation and searching for an effect equal to the standard deviation. According to this estimation, 15 animals were necessary in each group (GRAMNO 7.12, Barcelona, Spain). Results are shown as means and their standard deviations. The normal distribution of data was analyzed according to the Shapiro–Wilk test, and homology of variance among groups using Bartlett or Levene tests. Parameters fitting both criteria were compared using one-way ANOVA, according to Bonferroni multiple comparison test as post hoc analysis. Non-parametric Kruskal–Wallis ANOVA followed by Dunn’s multiple comparison was used to compare the groups failing in any of the hypotheses. Association between variables was assessed by Spearman’s correlation coefficient (ρ). All calculations were performed using SPSS version 15.0 software (SPSS Inc, Chicago, IL, USA) or Prism 5 for Windows (GraphPad, S. Diego, CA, USA). Significance was set at *p* ≤ 0.05.

## 3. Results

### 3.1. Composition of Experimental Diets

All purified diets designed to contain the same amount of macronutrients (Table 1) differed in the source of protein. Their nitrogen analyses confirmed that they were isonitrogenous. Consequently, their protein content amino acid composition was different as shown in Table 2. In this regard, the soybean-containing diet provided a lower amount of sulfur amino acids (0.16% cysteine and 0.10% methionine) in comparison with the other prepared diets (0.14 and 0.22% for cysteine and methionine, respectively in both cases). Compared with the avian diets, the amount of branched chain amino acids (BCAA) was also 20% lower in the soybean diet, with values of a 0.45% for valine, a 0.39% for isoleucine and a 0.66% for leucine.

### 3.2. Somatometric Analyses

During the dietary intervention, the three experimental groups showed similar body weight gains with independence of sex (Supplementary Figure S1, panels A and B). Nor were there statistically significant differences observed in solid intake among groups in either sex (Supplementary Figure S1, panels C and D).

### 3.3. Plasma Parameters

Table 3 reflects the studied analytes according to sex. In males, no significant changes were observed regarding plasma total cholesterol. Triglycerides were significantly (*p* < 0.05) higher in the group receiving the soybean than in the chicken and turkey diets. Between the groups consuming the avian diets, those receiving chicken protein showed a statistically significant lower value.

Glucose, NEFA and APOA1 concentrations did not experienced significant changes among studied conditions. PON1 activity was significantly (*p* < 0.05)) higher in the groups consuming the avian proteins than in the soybean group.

In females, no statistically significant changes were observed for total cholesterol, triglycerides, glucose and APOA1 among groups (Table 3). PON1 activity was significantly (*p* < 0.05) higher in the turkey than in the chicken, but no significant differences were observed between chicken and soybean groups.

The finding that total cholesterol did not show any significant changes among experimental groups both in males and females (Table 1) was corroborated when the distribution of total cholesterol carried in lipoproteins was assayed by FPLC profile (Table 4 and Supplementary Figure S2, panels A and B). However, changes in distribution of esterified and non-esterified cholesterol were observed between sexes and among experimental groups in function of sex. In this sense, the values of esterified cholesterol were higher in females than in males in all studied groups as shown in Table 4 and Supplementary Figure S2C,D, and females consuming the avian proteins showed significant decreases in VLDL and LDL esterified cholesterol. With respect to non-esterified cholesterol (Table 4 and Supplementary Figure S2E,F), females consuming the avian proteins showed a significant increase in VLDL and LDL with respect to soybean group. The males consuming the different source of protein did not display such profound changes (Table 4).

### 3.4. ROS

**Figure 1:**
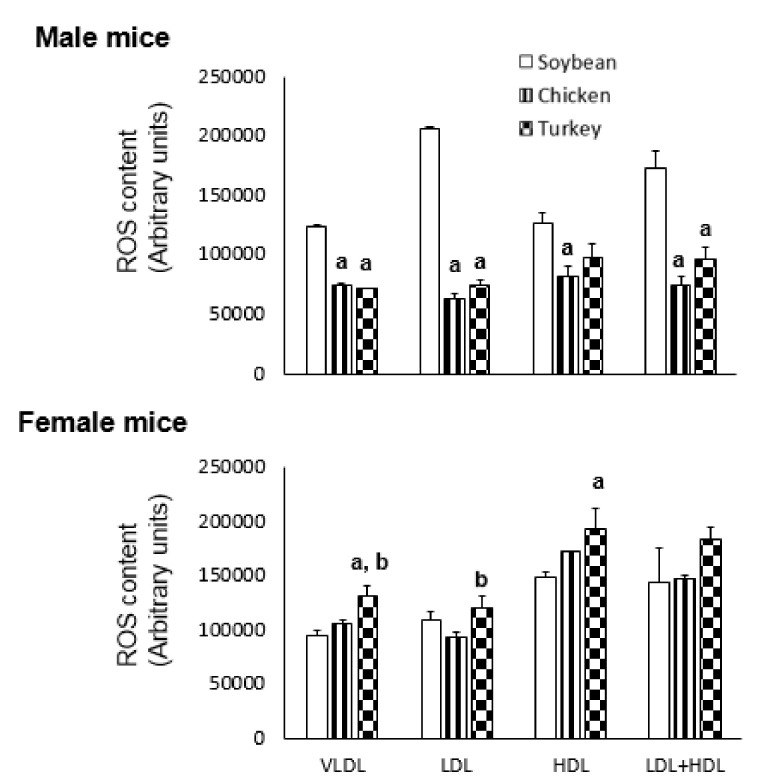
Effect of different protein sources on lipoprotein ROS content in APOE KO mice. ROS levels in the lipoprotein fractions from different groups were assayed using 2,7-dichlorofluorescein diacetate for 24 h and expressed as arbitrary fluorescence units. Each pool was assayed in triplicate and the results are shown as means ± SD. Statistical analyses were carried out by one-ANOVA and Bonferroni post-hoc test. ^a^, *p* < 0.05 vs. soybean and ^b^, *p* < 0.05 vs. chicken.

The oxidative stress present in different FPLC-isolated lipoproteins was assayed by incubation in presence of dichlorofluorescein and the results are shown in Figure 1. As reflected in Figure 1, males consuming the avian proteins showed significantly less ROS content in VLDL and LDL than those receiving the soybean protein diet. In contrast, females consuming turkey protein showed significantly higher ROS content in VLDL, LDL and HDL than the soybean group (Figure 1).

### 3.5. Aortic Atherosclerotic Lesions

**Figure 2:**
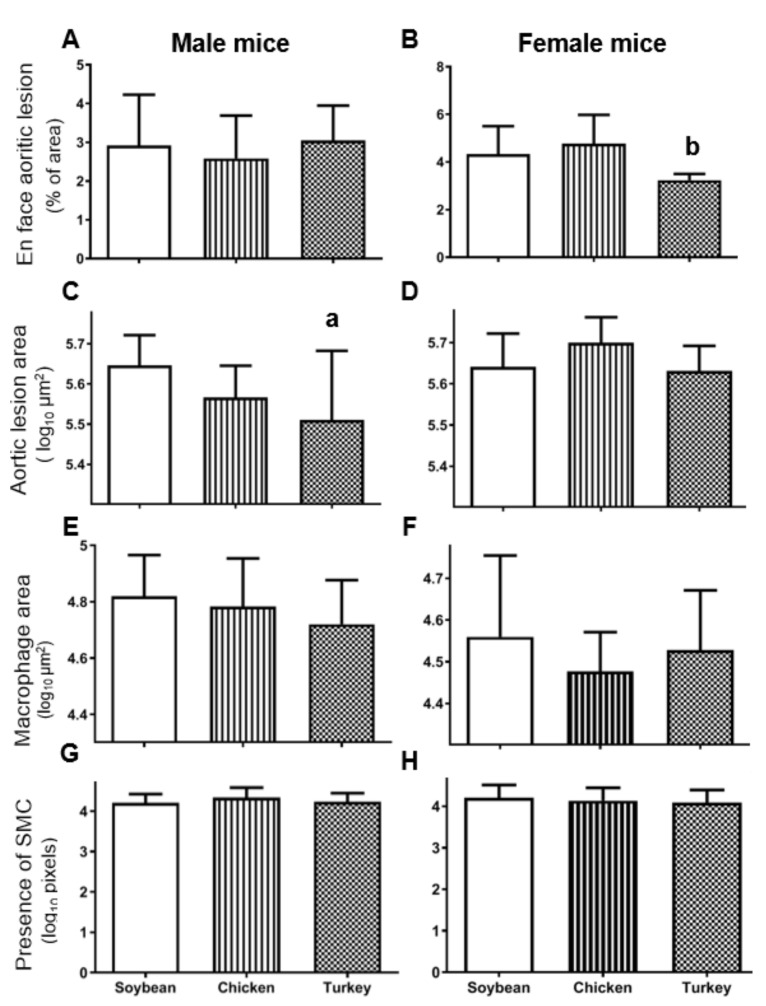
Atherosclerosis lesions in APOE KO mice consuming the different diets. En face aortic analyses of whole aortas expressed as percentage of total area occupied by lesions (**A**,**B**, males and females, respectively). Cross sectional lesion areas at the aortic root analyzed by oil red staining and expressed as log_10_ µm^2^ (**C**,**D**). Lesion areas covered by macrophages according to CD68 immunostaining expressed as log_10_ µm^2^ (**E**,**F**). Presence of smooth muscle cells immunostaining for α-actin expressed as arbitrary pixel areas (**G**,**H**). The results are shown as means and SD. Statistical analyses were carried out by ANOVA followed by Bonferroni post-hoc test. ^a^, *p* < 0.05 vs. soybean and ^b^, *p* < 0.05 vs. chicken.

The en face analyses of aortas shown in Figure 2A corresponded to males in the different experimental groups where no statistically significant change was observed. In contrast, the same analyses carried out in females (Figure 2B) evidenced a significant decrease (*p* < 0.05) in atherosclerotic foci in the group receiving the turkey protein diet when compared with the chicken group.

When cross-sectional analyses of aorta were performed, male mice receiving the turkey protein diets displayed statistically significant decreased lesion areas than the group fed with soybean protein (Figure 2C and Supplementary Figure S3A–C). In contrast, this decrease was not observed in females consuming the turkey protein diet (Figure 2D and Supplementary Figure S3D–F). The characteristics of the atherosclerotic lesions were evaluated by immunostaining of macrophages and smooth muscle cells. The former, evaluated as CD68 immunostaining, did not change significantly among studied groups in either sex (Figure 2E,F). The presence of smooth muscle cells was revealed by using anti-α actin staining and expressed as its covered lesion area. As shown in Figure 2G,H, no significant changes were evidenced by different diets in both sexes.

Regarding calcification foci, they were only observed in one animal per each group, suggesting that our diets without cholate [32] are very mild to induce this atherosclerotic complication.

### 3.6. Hepatic Steatosis

Quantitative analyses of hepatic lipid droplets (Supplementary Figure S4) did not show any statistical difference among the studied dietary groups in either sex.

### 3.7. Association Studies

**Figure 3:**
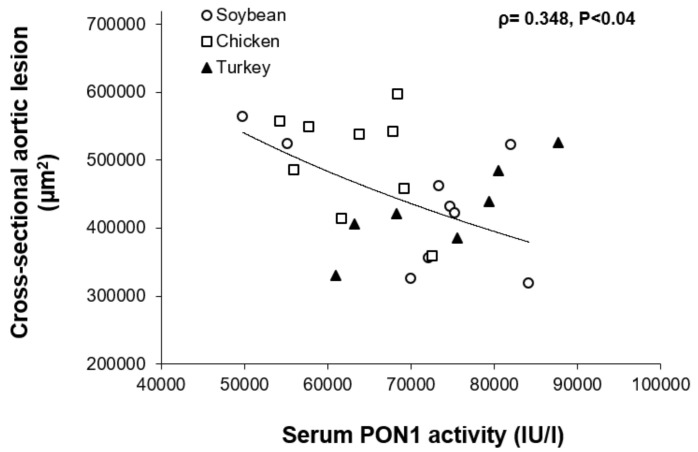
Relationship between serum paraoxonase activity and cross-sectional aortic lesions in female individual mice from all dietary groups. Spearman’s rank-order correlation coefficient (ρ) and its probability are shown.

A significant inverse association was found between PON1 activity and cross-sectional aortic lesion in females (Figure 3).

## 4. Discussion

The present investigation was carried out to study the influence of three dietary protein sources on atherosclerotic development and hepatic steatosis and its associated plasma risk factors in male and female *Apoe*-deficient mice fed Western diets. Three diets were prepared to provide the same amount of protein as well as other macronutrients. Amino acid composition showed differences in sulfur amino acids (methionine and cysteine) and BCAA (isoleucine, leucine and valine), that were lower in the soybean diet (Table 2). In this way, the changes in amino acid composition were the main variables among the three different diets. There were statistically significant sex differences in the response to the diets regarding triglycerides, esterified cholesterol levels and ROS in lipoproteins. Likewise, only females consuming the turkey protein-containing diet showed significantly decreased atherosclerotic foci, as evidenced by the en face atherosclerosis analyses. The presence of macrophages, according to CD68 staining, and SMC were not modified, nor were hepatic lipid droplets in any of the studied groups. PON1 activity was significantly higher in the group consuming turkey protein without sex differences. Overall, these findings suggest a complex interaction of dietary proteins and sex on plasma lipid composition and function, and these on the initiation and growth of atherosclerotic plaques.

According to our results, the provided amounts of BCAA in the soybean group were slightly lower than the required by mice [33]. Despite this fact, parameters related to insulin resistance such as body weight, fatty liver, glucose and NEFA were not found significantly influenced by soybean protein when compared with the groups receiving the avian proteins. In this regard, glycine has been proposed as the amino acid involved in the BCAA action [34], and the lower levels of glycine in the soybean diet could explain the absence of changes in the present experimental setting. Only males consuming soy protein showed elevated triglycerides, this could be due to the lack of effect in this sex of the bioactive phyto compounds (flavonoids, polyphenols, phytoestrogens, etc.) in the ~10% of soy protein isolate provided at 0.1% of ingested diet and in a dose of 100 mg/kg. The relevance of soy protein has also been shown by Dhot et al. regarding diastolic dysfunction [35].

Particularly relevant was the sex differences in the esterified cholesterol carried in lipoproteins and the statistically significant decrease that avian proteins exerted. This observed effect in an animal model lacking cholesterol ester transfer protein [36] as is *Apoe*-deficient mouse, should be indicative of higher hepatic loading and the latter modified by nature of dietary protein in a sex-dependent way. While the hepatic loading of triglycerides into APOB-containing particles has been extensively studied [37], the mechanisms involved in the loading of cholesterol and regulation are poorly understood and an involvement of certain amino acids cannot be rejected.

Previous studies tackling the effect of soy protein on atherosclerosis did not address the sex difference either by combining results [11] or by using ovariectomized females [12,13]. In these studies, the amount of protein provided to mice was 20%. Our results clearly indicate that sex should be taking into consideration since plasma parameters such as triglycerides, non-esterified cholesterol levels and ROS in lipoproteins experienced significant different responses according to sources of protein and the changes were even observed with a lower protein supply (11.2% of the current study).

Our evaluation of the atherosclerosis lesion indicates that the source of protein has an important impact on atherosclerosis extent, growth and characteristics of atherosclerotic plaques. This information was not considered in previous studies addressing the anti-atherosclerotic properties of soybean proteins since they analyzed in a subrogate way using aortic cholesterol content [11,12,13]. Only the work published by Sawashita et al. observed a decrease in atherosclerotic foci when soybean protein was compared with α-casein as source of protein and fed to male double *Apoe* and *Ldlr* deficient mice [16]. In the latter study, chicken protein showed a similar atherosclerotic effect when compared to soybean proteins, something that has been confirmed in the current work. Our results clearly evidence that sex is crucial in the observed responses, with females being more responsive to turkey protein-containing diets in terms of extent of atherosclerotic foci (Figure 2B), without differences in growth of plaques and presence of macrophages and smooth muscle cells (SMC). The latter aspects are involved in plaque stabilization [38]. Macrophages as etiological source of proteases able to degrade the cap fibers and SMC biosynthesizing the cap fibers were not found to change by the source of administered protein.

Taken together, in all data of males and females in the different experimental groups, a significant association between en face and cross-sectional lesion data was found (ρ = 0.32, *p* < 0.01). This in agreement with our results reported previously (ρ = 0.33) in other nutritional interventions [39]. Collectively, the presence of new atherosclerotic foci evaluated by en face analysis involves different cellular and molecular mechanisms of those taking place in growth of existing plaques, evaluated by the cross-sectional method. While, in the former, endothelial cells are primarily executers of lipoprotein and monocyte recruitments, the growth of a plaque is more complex since not only those processes are involved, but in addition, differentiation of monocytes into macrophages, proliferation, metabolism and necrosis of macrophages and proliferation and metabolism of SMC play important roles [40].

A remarkable finding of our study is the selective action of turkey source of protein on certain parameters such as PON1 activity that was particularly higher when compared with other diets and without sex differences. However, its association with the cross-sectional aortic lesion was only statistically significant in females. This is contrast with what we observed in other experimental settings, where the association was stronger in males than in females and when dietary cholesterol was variable [39]. In our present study, dietary cholesterol was kept constant. However, the observed changes cannot be attributed to amino acid composition itself since not much difference was observed between the used avian proteins. In this sense, previous experience with peptides of other protein sources such as soybean [13], fish [41], chick pea [41] or fenugreek [42] has proved to be active regarding lipoprotein metabolism. In this way, post-translational modifications of certain amino acids altering the digestive pattern of these avian proteins and generation of specific peptides with biological properties is a suggestive hypothesis that needs to be tested in future experiments. The correlation between PON1 activity and atherosclerotic corroborates previous results in mice [43] and in humans [44], and related to Mediterranean Diet components [45]. The observed lipoprotein ROS content (Figure 1), which showed a greater oxidation of LDL in the soybean group, was in agreement with the PON1 results, particularly in males. The arylesterase activity of paraoxonase is used as a surrogate marker of the amount of circulating PON1, an enzyme associated with HDL [45]. HDL is currently estimated by its cholesterol content which is only a measurement of HDL particles loaded with cholesterol [46,47]. The groups fed the chicken protein had higher HDL, but lower PON, this could be due to a PON1 particularly linked to lipid-poor HDL. In general, the correlation between PON1 and HDL cholesterol levels only existed in males (ρ = 0.25, *p* < 0.05). This value of correlation even improved (ρ = 0.318, *p* < 0.017) when esterified cholesterol was used. Since esterified cholesterol is representative of larger HDL particles once they have taken up cholesterol and lecithin: cholesterol acyltransferase has esterified it, this would suggest that some the larger HDL carry PON1. Interestingly, in males, no significant association was seen between PON1 and APOA1 (ρ = 0.118, *p* < 0.2). These results are suggestive that PON1 is carried in HDL lipoparticles not containing APOA1 as we previously observed in other nutritional settings where specifically HDL containing APOA4 were involved [48]. Overall, the nutritional regulation of paraoxonase is sex dependent and executed through HDL lipoparticles and their metabolism. The specific involvement of amino acids or their peptides on the regulation of PON1 in a sex-dependent way is an interesting aspect that deserves further attention.

## 5. Conclusions 

The consumption of avian proteins resulted in a similar outcome compared to the soybean source of protein regarding the presence of fatty liver, macrophages and SMC in atherosclerotic plaques. However, in aspects such as growth of atherosclerotic plaques, only females consuming the turkey protein-containing diet showed significantly decreased atherosclerotic foci. Metabolic parameters such as triglycerides, esterified cholesterol levels and ROS in lipoproteins are influenced by dietary source of protein in a sex-dependent way. PON1 activity was significantly higher in the group consuming turkey protein without sex differences, but its association with cross-sectional atherosclerosis was only observed in females. Therefore, studying the biological properties of dietary proteins, sex is a critical factor that should definitively be taken into consideration.

## Figures and Tables

**Table 1:** Composition of experimental diets.

Ingredients	Soybean (%, *w/w*)	Chicken (%, *w/w*)	Turkey (%, *w/w*)
Corn starch ^a^	37.2	37.2	37.2
Soybean protein	11.2		
Chicken protein		11.2	
Turkey protein			11.2
Maltodextrin ^a^	12.4	12.4	12.4
Sucrose ^b^	8.0	8.0	8.0
Soybean oil ^c^	3.2	3.2	3.2
Cellulose ^d^	4.0	4.0	4.0
Mineral mix ^e^	2.8	2.8	2.8
Vitamin mix ^f^	0.8	0.8	0.8
Choline ^g^	0.2	0.2	0.2
L-Cystine ^g^	0.1	0.1	0.1
Cholesterol ^g^	0.15	0.15	0.15
Palm oil ^h^	20	20	20

Based on AIN-93 recommended diets for laboratory rodents [22]. ^a^ Cargill, Barcelona, Spain. ^b^ Azucarera Ibérica S.L., Madrid, Spain. ^c^ Aceites Muñoz, Toledo, Spain. ^d^ Vitacel R200, Rettenmaier Ibérica S.L, Barcelona, Spain. ^e^ AIN-93M Mineral Mix (MP Biomedicals, Illkirch, France). The salt mixture contains the following amounts (g/kg): calcium carbonate, 357; monopotassium phosphate, 250; potassium citrate monohydrate, 28; sodium chloride, 74; potassium sulphate, 46.6; magnesium oxide, 24; ferric citrate, 6.06; zinc carbonate, 1.65; manganese carbonate, 0.63; copper carbonate, 0.3; potassium iodate, 0.01; sodium selenate anhydrous, 0.01025; ammonium molybdate.4H_2_O, 0.00795; sodium metasilicate.9H_2_O, 1.45; chromium potassium sulfate.12H_2_O, 0.275; lithium chloride, 0.0174; boric acid, 0.0815; sodium fluoride, 0.0635; nickel carbonate, 0.0318; ammonium vanadate, 0.0066 and powdered sugar, 209.806. ^f^ AIN-93-VX Vitamin Mix (MP Biomedicals, Illkirch, France). Vitamin mixture contains the following amounts (mg/kg): nicotinic acid, 3; D-calcium pantothenate, 1.6; pyridoxine HCl, 0.7; thiamine HCl, 0.6; riboflavin, 0.6; folic acid, 0.2; D-biotin, 0.02; vitamin B_12_ (0.1% triturated in mannitol), 2.5; α-tocopherol powder (250 U/g), 30; vitamin A palmitate (250,000 U/g), 1.6; vitamin D_3_ (400,000 U/g), 0.25; phylloquinone, 0.075 and powdered sucrose, 959.655. ^g^ Sigma-Aldrich Química, Madrid, España. ^h^ Gustav Heess, Barcelona, Spain.

**Table 2:** Total protein and amino acid composition of the experimental diets.

	Soybean	Chicken	Turkey
Protein (%, *w/w* dry)	10.9 ± 0.2	10.6 ± 0.1	10.7 ± 0.1
Amino acids (%, *w/w*) *			
Non-essential			
Alanine (Ala)	0.40	0.61	0.62
Arginine (Arg)	0.64	0.59	0.61
Aspartic acid (Asp)	1.00	0.93	1.00
Cysteine (Cys)	0.16	0.14	0.14
Glutamic acid (Glu)	1.66	1.46	1.47
Glycine (Gly)	0.36	0.44	0.43
Proline (Pro)	0.46	0.38	0.36
Serine (Ser)	0.46	0.41	0.39
Tyrosine (Tyr)	0.29	0.27	0.30
Total non-essential	5.43	5.23	5.32
Essential			
Histidine (His)	0.20	0.28	0.29
Isoleucine (Ileu)	0.39	0.49	0.48
Leucine (Leu)	0.66	0.81	0.79
Lysine (Lys)	0.58	0.89	0.90
Methionine (Met)	0.10	0.22	0.22
Phenylalanine (Phe)	0.44	0.32	0.31
Threonine (Thr)	0.34	0.44	0.44
Tryptophan (Trp)	0.15	0.13	0.11
Valine (Val)	0.45	0.55	0.55
Total essential	3.31	4.13	4.09
Essential/Non-essential	0.6	0.8	0.8

Data are means ± SD for each group. Statistical analysis was carried out by ANOVA followed by Bonferroni post-hoc test. * Results are shown as average.

**Table 3:** Plasma parameters.

	Soybean Group	Chicken Group	Turkey Group
Males (*n*)	14	15	16
Triglycerides (mg/dL) *	150 ± 35	88 ± 17 ^a^	115 ± 44 ^a,b^
Total cholesterol (mg/dL)	808 ± 128	765 ± 166	670 ± 230
Glucose (mg/dL)	277 ± 41	220 ± 44	342 ± 182
Non-esterified fatty acids (mg/dL)	37 ± 6	44 ± 8	38 ± 13
Apolipoprotein A1 (arbitrary units)	4.1 ± 0.6	3.9 ± 0.5	4.2 ± 0.6
Paraoxonase 1 (IU/L) *	43,249 ± 5704	62,541 ± 10,589 ^a^	74,940 ± 17,834 ^a^
Females (*n*)	10	10	10
Triglycerides (mg/dL)	168 ± 35	177 ± 44	212 ± 71
Total cholesterol (mg/dL)	510 ± 163	553 ± 93	502 ± 58
Glucose (mg/dL)	270 ± 71	320 ± 95	341 ± 79
Non-esterified fatty acids (mg/dL)	43 ± 8	44 ± 7	44 ± 10
Apolipoprotein A1 (arbitrary units)	5.0 ± 0.6	4.6 ± 0.4	4.7 ± 0.5
Paraoxonase 1 (IU/L)	70,076 ± 10,891	63,461 ± 6493	74,854 ± 11,778 ^b^

Data are means ± SD for each group. Unless specified, statistical analysis was carried out by ANOVA followed by Bonferroni post-hoc test. * Statistical analysis was carried out by non-parametric Kruskal–Wallis ANOVA followed by Dunn’s multiple comparison test. ^a^, *p* < 0.05 vs. soybean and ^b^, *p* < 0.05 vs. chicken.

**Table 4:** Distribution of cholesterol in plasma lipoproteins.

	Soybean Group	Chicken Group	Turkey Group
Males (*n*)	14	15	16
Total cholesterol (mg/dL)			
VLDL	510 ± 92	447 ± 129	465 ± 200
LDL	272 ± 49	239 ± 70	248 ± 107
HDL	48 ± 9	42 ± 12	44 ± 14
Esterified cholesterol (mg/dL)			
VLDL	121 ± 22	106 ± 31	79 ± 34 ^a,b^
LDL	96 ± 17	84 ± 24	82 ± 35
HDL	34 ± 6	30 ± 8	33 ± 14
Non-esterified cholesterol (mg/dL)			
VLDL	396 ± 71	347 ± 100	384 ± 161
LDL	166 ± 30	146 ± 42	161 ± 69
HDL	16 ± 3	14 ± 4	15 ± 7
Females (*n*)	10	10	10
Total cholesterol (mg/dL)			
VLDL	337 ± 58	352 ± 59	279 ± 55 ^a,b^
LDL	179 ± 31	175 ± 29	180 ± 35
HDL	15 ± 3	25 ± 4 ^a^	18 ± 4 ^b^
Esterified cholesterol (mg/dL)			
VLDL	224 ± 39	92 ± 16 ^a^	109 ± 21 ^a^
LDL	144 ± 25	80 ± 13 ^a^	92 ± 18 ^a^
HDL	8 ± 1	13 ± 2 ^a^	9 ± 2 ^b^
Non-esterified cholesterol (mg/dL)			
VLDL	112 ± 19	261 ± 44 ^a^	191 ± 37 ^a,b^
LDL	36 ± 6	94 ± 16 ^a^	69 ± 14 ^a,b^
HDL	7 ± 1	10 ± 2 ^a^	7 ± 1 ^b^

Data are means ± SD for each group. Fractions were prepared by FPLC and their cholesterol assayed. Statistical analysis was carried out by ANOVA followed by Bonferroni post-hoc test. ^a^, *p* < 0.05 vs. soybean and ^b^, *p* < 0.05 vs. chicken. HDL, high density lipoproteins, LDL, low density lipoproteins and VLDL, very low density lipoproteins.

## Data Availability

Data will made available to authors on reasonable request.

## Supplementary Materials

The following are available online at https://www.mdpi.com/article/10.3390/nu13061838/s1, Figure S1: Follow-up of body weight during diet intervention in males (A) and females (B) and solid intake in males (C) and females (D). Data are means ± SD for each group. Statistical analyses were carried out by ANOVA followed by Bonferroni post-hoc test; Figure S2: Effects of different diets on plasma lipoproteins. Representative FPLC profile of collected fractions analyzed for total cholesterol (A,B), esterified cholesterol (C,D) and non- esterified cholesterol (E,F) in males and females, respectively. APOEKO mice received the different diets during 12 weeks. Fraction numbers 1–6 corresponded to VLDL/chylomicron remnants, 7–13 to low density lipoproteins, 14–18 to cholesterol-rich HDL and 19–24 to cholesterol-poor HDL; Figure S3: Atherosclerosis lesions in APOE KO mice consuming the different diets. Representative cross sections of the aortic roots stained with oil red from males consuming soybean (A), chicken (B) and turkey (C) source of proteins and females consuming soybean (D), chicken (E) and turkey (F) source of proteins; Figure S4: Effect of the diets on hepatic fat in APOE KO mice. Results are shown as means ± SD for each group. Statistical analyses were carried out by ANOVA followed by Bonferroni post-hoc test.

## Abbreviations

APOA1	apolipoprotein A1
BCAA	branched-chain amino acids
FPLC	fast performance liquid chromatography
HDL	high density lipoproteins
LDL	low density lipoproteins
NEFA	non-esterified fatty acids
PON1	paraoxonase
ROS	reactive oxygen species
VLDL	very low density lipoproteins
