# Supplementary material for: Dietary Avian Proteins Are Comparable to Soybean Proteins on the Atherosclerosis Development and Fatty Liver Disease in Apoe-Deficient Mice
Source: Nutrients. 2021 May 27;13(6):1838. doi: 10.3390/nu13061838 (PMC8227708; doi:10.3390/nu13061838)
Supplement: Supplementary file 1 [file nutrients-13-01838-s001.zip › nutrients-1215370-supplementary.pdf]

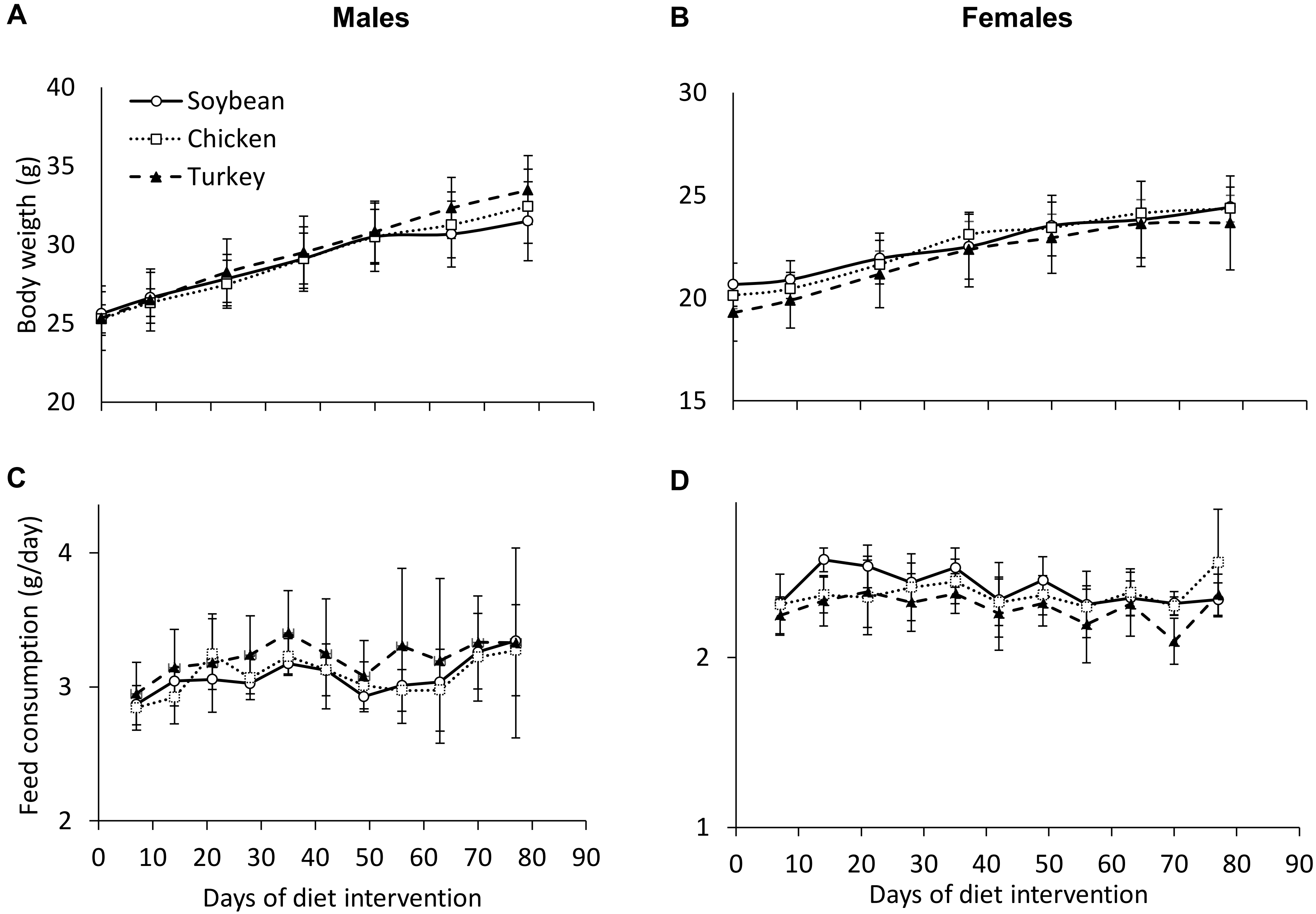

Supplemental Figure 1. Follow-up of body weight during diet intervention in males (A) and females (B) and solid intake in males (C) and females (D). Data are means ± SD for each group. Statistical analyses were carried out by ANOVA followed by Bonferroni post-hoc test.

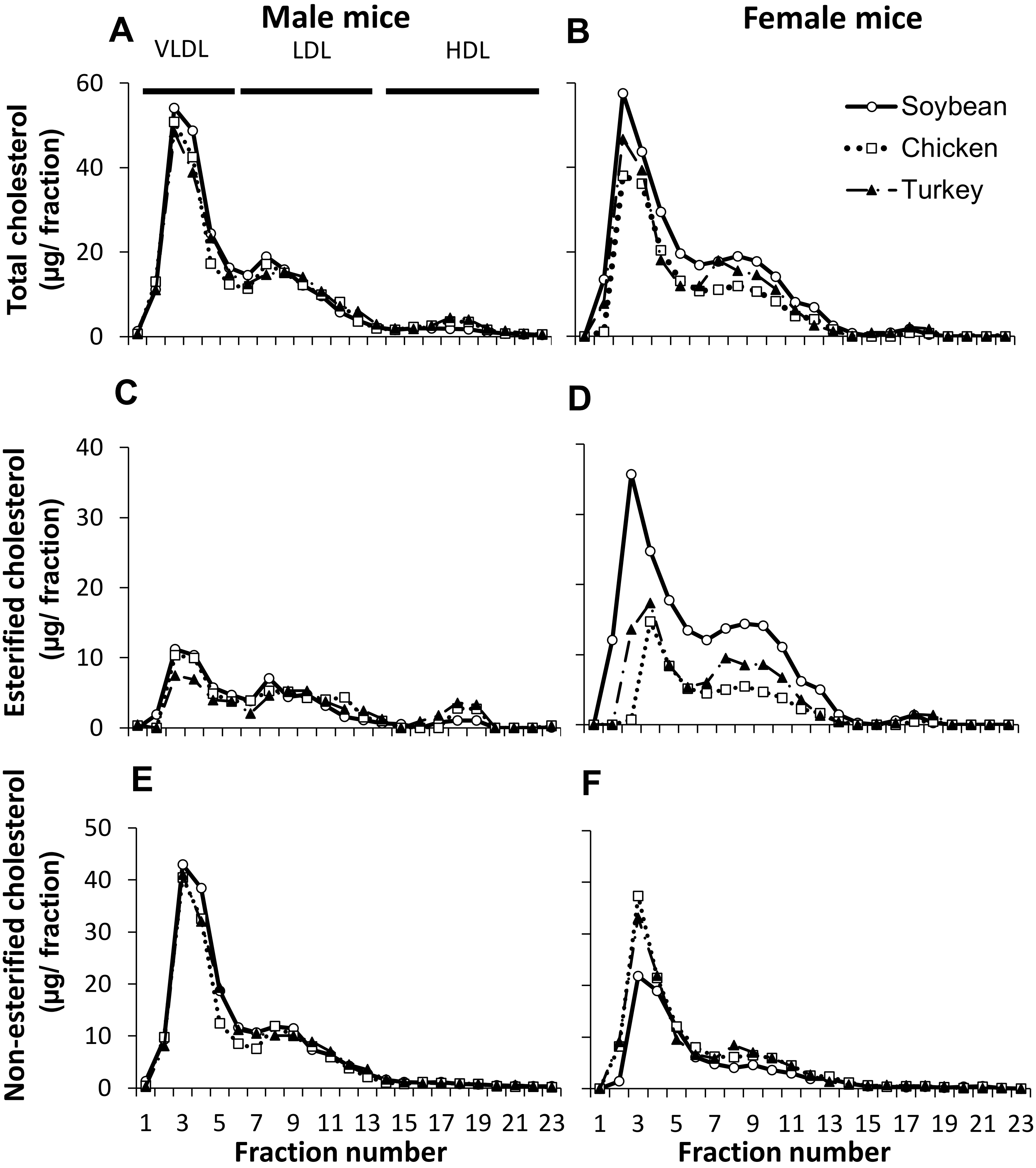

Supplementary Figure 2. **Effects of different diets on plasma lipoproteins.** Representative FPLC profile of collected fractions analyzed for total cholesterol (A and B), esterified cholesterol (C and D) and non-esterified cholesterol (E and F) in males and females, respectively. APOE KO mice received the different diets during 12 weeks. Fraction numbers 1–6 corresponded to VLDL/chylomicron remnants, 7–13 to low density lipoproteins, 14–18 to cholesterol-rich HDL and 19–24 to cholesterol-poor HDL.

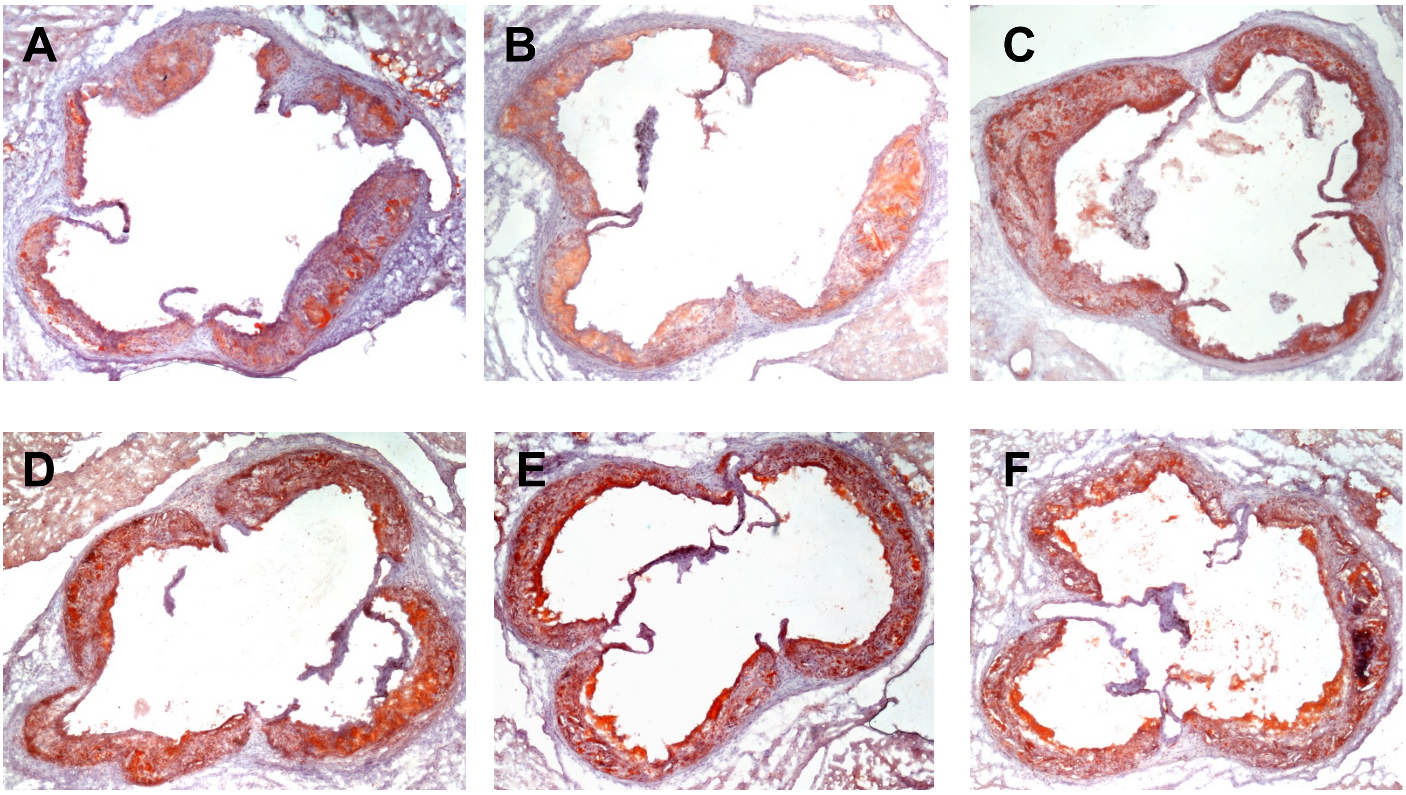

Supplemental figure 3. Atherosclerosis lesions in APOE KO mice consuming the different diets.

Representative cross sections of the aortic roots stained with oil red from males consuming soybean (A), chicken (B) and turkey (C) source of proteins and females consuming soybean (D), chicken (E) and turkey (F) source of proteins.

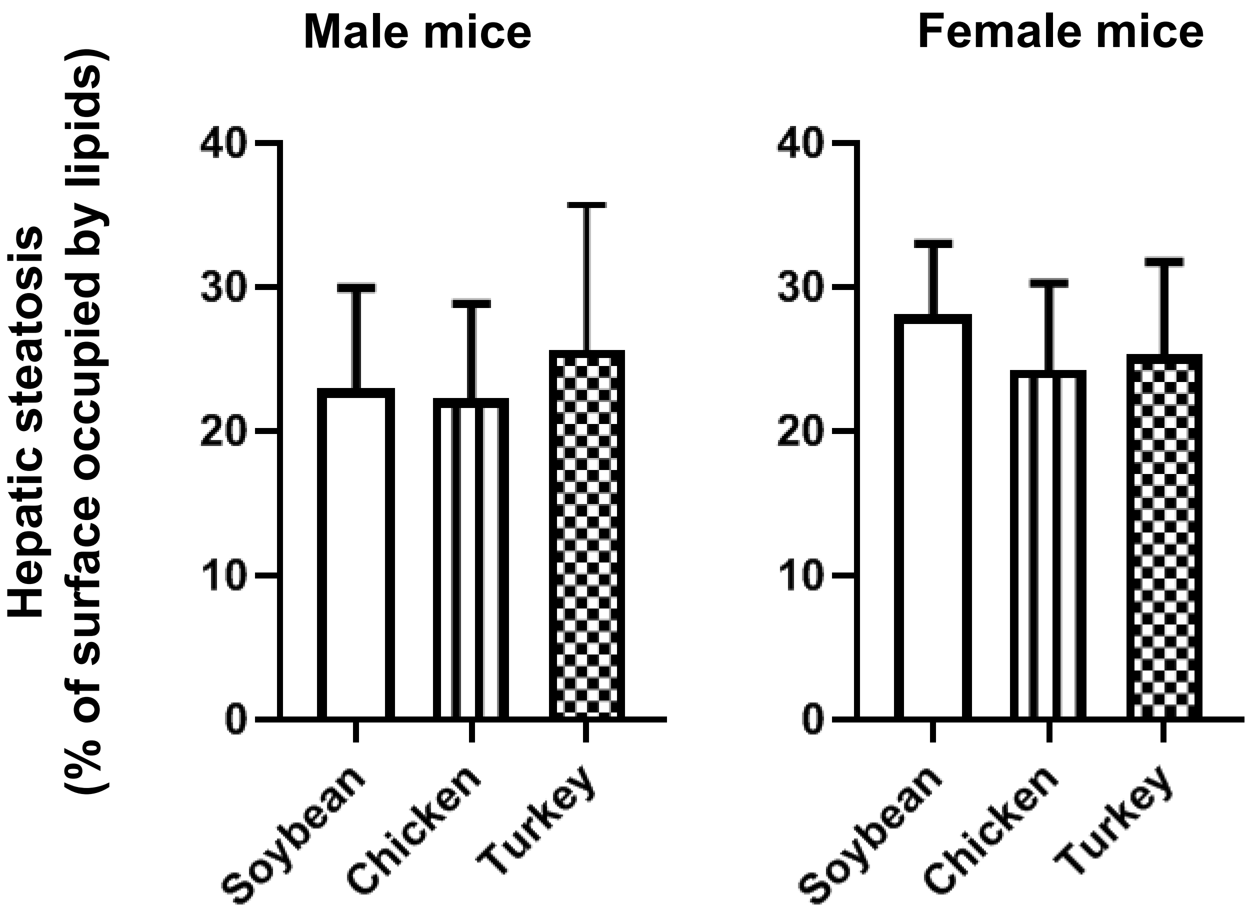

Supplemental figure 4. **Effect of the diets on hepatic fat in APOE KO mice.** Results are shown as means ± SD for each group. Statistical analyses were carried out by ANOVA followed by Bonferroni post-hoc test.
